# Biology by Numbers—Introducing Quantitation into Life Science Education

**DOI:** 10.1371/journal.pbio.0030001

**Published:** 2005-01-18

**Authors:** Tinri Aegerter-Wilmsen, Ton Bisseling

## Abstract

An online educational module introduces students to concepts of quantitation and numerical simulations in developmental biology

Driven by the massive datasets that are generated by “omics” research, the molecular life sciences are entering a new phase. This phase is characterised by a shift in focus from individual genes and their products to networks and whole systems [1,2,3]. For a thorough analysis of the behaviour of networks and their underlying principles, quantitative tools are often necessary. Numerical simulations can, for example, be used to explore the behaviour of a network when the values of different parameters are varied, and, in turn, mathematical analysis can help to understand a particular biological phenomenon [2].

The successful application of quantitative tools in the molecular life sciences requires a good understanding of these tools and sufficient knowledge of the biological system under study. This can be achieved by collaboration between quantitatively trained scientists such as physicists on the one hand and biologists on the other. However, cultural differences hamper such collaboration [1]: even at the undergraduate level, students in the different disciplines speak very different languages [4].

A more productive approach is therefore to prepare students better for the quantitative nature of the molecular life sciences by integrating quantitative thinking and biology in the life science curriculum. This can be achieved in various ways. For example, a curriculum could be developed in which mathematics, the physical sciences, and biology are introduced together [4]. However, we recommend that quantitative thinking also be included throughout the curriculum in the biology courses themselves, covering topics such as cell biology, developmental biology, and biochemistry. We consider this important because it will help to show students how quantitative tools can be used to address various cutting edge questions in biology.

## A Modelling Module in Developmental Biology

As an example of the integration of quantitative teaching and cutting edge biology, we have implemented an educational module in which numerical simulations are used in an existing course on developmental biology (http://mbedu.fbt.eitn.wau.nl/demo_plos/). Some of the features of this module and the thinking that led to its development are quite general, and so we present the module here as a case study in the hope that this might inspire and guide others to create similar resources.

First, we wanted to illustrate to students the value of using numerical simulations to study a developmental process. Therefore, a pattern-forming mechanism was selected that can initially be rather hard to understand: the generation of the morphogen gradient formed by the extracellular signalling molecule decapentaplegic (Dpp) early during Drosophila embryogenesis [5]. The generation of this gradient results from the fact that key proteins are synthesized in different embryonic regions, from the formation of complexes of these proteins, and from the different diffusion rates of these complexes and their components, as well as from the specific degradation of some components. Students are guided through the creation of a model for Dpp gradient formation based on a set of experimental data. At several stages, students can perform simulations in a separate simulation environment. Students use simulations, for example, to check whether a number of core interactions is sufficient to yield the most important characteristics of the wild-type gradient.

**Figure 1:**
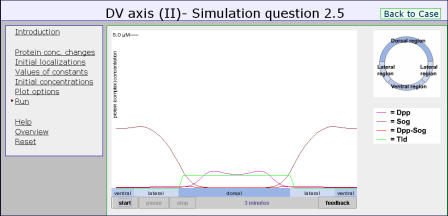
A Simulation That Students Can Perform After several minutes, Dpp forms one peak in the centre of the dorsal region, as in the wild type. The various elements of the quantitative model can be entered under “protein conc. changes”, “initial localizations”, “values of constants”, and “initial concentrations”. The numerical simulation itself shows the dynamic behaviour of the designed quantitative model.

**Figure 2:**
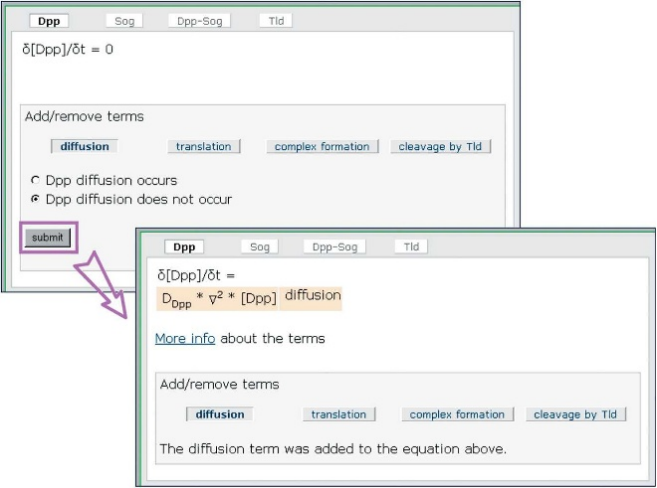
Illustration of How Students Can Set Up Differential Equations If a student indicates that Dpp diffusion occurs, a diffusion term is added to the differential equation that describes the changes in Dpp concentration.

Second, we designed the simulation environment in such a way that biology students with their existing mathematical background can build quantitative models and run numerical simulations themselves. In this environment (Figure 1), students do not have to program anything, or set up differential equations, themselves. Instead, they indicate which processes occur at the molecular level, and the program then shows how each of these processes is translated into a term in a differential equation. In Figure 2, for example, the program adds a diffusion term to a differential equation if the student indicates that diffusion occurs. Besides setting up the equations in this way, students specify the initial localisations and concentrations of the different proteins, as well as the constants that are used in the differential equations.

Third, we wanted to make sure that students would use the simulation environment effectively. Therefore, a clear goal is formulated when students enter the simulation environment. For example, they are asked to make a model that generates a Dpp gradient that fulfills a number of specific criteria, or simulates certain mutants. After running a simulation, students can view feedback that helps them draw conclusions or consider the next step to be taken. If a student's model, for example, generates a gradient that is too shallow, the student has to indicate which change in the model he expects to be useful for generating a steeper gradient. The student then receives an intuitive explanation of the usefulness of the given suggestion. If an increase in the synthesis of one of the proteins, Short gastrulation, is proposed, for example, feedback is given that this could indeed be useful, since there would then be more Short gastrulation available to transport Dpp, such that the gradient can become steeper. In this way, the student is stimulated to carefully consider each step and is provided with sufficient support to decide which is a useful step to follow. In addition, with this type of feedback, explanations are given that relate quantitative changes in the model to qualitative changes in its behaviour, which should increase the student's understanding of the behaviour of the biological model.

We consider it important that students, while using the module, are not distracted too much by quantitative issues from the actual biological principles and facts. These have to be mastered in order to obtain a strong biological background. If students want to learn more advanced quantitative skills, they can still follow courses that are specifically aimed at this aspect.

## The Future

Quantitative analysis is already gaining importance in molecular life sciences. Therefore, it is desirable that curriculum changes are implemented in the short term. This poses challenges to faculties, especially to those whose members do not have much, if any, experience with the application of quantitative tools in their own research. Therefore, it may be useful to initially focus on the development of learning materials that are rather self-contained, such that their application requires relatively little competence in quantitative analysis from the teaching staff. If these materials are openly available they can be incorporated rapidly into existing courses, such that even the current generation of students may be better prepared to integrate quantitative thinking and biology in their future research.

## Abbreviation

Dpp: decapentaplegic

## References

[pbio-0030001-b1] Knight J (2002). Bridging the culture gap. Nature.

[pbio-0030001-b2] Lander AD (2004). A calculus of purpose. PLoS Biol.

[pbio-0030001-b3] Pennisi E (2003). Tracing life's circuitry. Science.

[pbio-0030001-b4] Bialek W, Botstein D (2004). Introductory science and mathematics education for 21st-century biologists. Science.

[pbio-0030001-b5] Eldar A, Dorfman R, Weiss D, Ashe H, Shilo BZ (2002). Robustness of the BMP morphogen gradient in Drosophila embryonic patterning. Nature.

